# Genotoxicity Assessment of Nutraceuticals Extracted from Thinned Nectarine (*Prunus persica* L.) and Grape Seed (*Vitis vinifera* L.) Waste Biomass

**DOI:** 10.3390/foods12061171

**Published:** 2023-03-10

**Authors:** Giorgia Musto, Elisabetta Schiano, Fortuna Iannuzzo, Gian Carlo Tenore, Ettore Novellino, Mariano Stornaiuolo

**Affiliations:** 1 Department of Pharmacy, University of Naples Federico II, Via Montesano 49, 80131 Naples, Italy; 2 Department of Medicine and Surgery, Catholic University of the Sacred Heart, 00168 Rome, Italy

**Keywords:** food waste, nutraceuticals, Ames test, genotoxicity, polyphenols, immature nectarines, grape seed extract, *Vitis vinifera*

## Abstract

Agri-food by-products represent a considerable portion of the waste produced in the world and especially when incorrectly disposed of, contribute to air, soil, and water pollution. Recently, recycling of food waste has proven to be an attractive area of research for pharmaceutical companies, that use agri-food by-products (leaves, bark, roots, seeds, second-best vegetables) as alternative raw material for the extraction of bioactive compounds. Developers and producers are however, advised to assess the safety of nutraceuticals obtained from biowaste that, in virtue of its chemical complexity, could undermine the overall safety of the final products. Here, in compliance with EFSA regulations, we use the Ames test (OECD 471) and the micronucleus test (OECD 487) to assess the mutagenicity of two nutraceuticals obtained from food waste. The first consists of grape seeds (*Vitis vinifera* L.) that have undergone a process of food-grade depolymerization of proanthocyanidins to release more bioavailable flavan-3-ols. The second nutraceutical product consists of thinned nectarines (*Prunus persica* L. *var nucipersica*) containing abscisic acid and polyphenols. The results presented here show that these products are, before as well as after metabolization, non-mutagenic, up to the doses of 5 mg and 100 μg per plate for the Ames and micronucleus test, respectively, and can be thus considered genotoxically safe.

## 1. Introduction

During food processing, more than 70% of the biological feedstock becomes waste. This percentage increases for highly consumed food products like palm oil, the main cooking oil in non-European countries, for which waste biomass can reach up to the 90% of the harvested fruits. Every year, one billion tons of food by-products are discarded worldwide, and this amount is set to rise even further within the next decades [1,2]. Food waste is a pollutant with a huge environmental impact and, considering the cost for its disposal, it represents a considerable global emergency [1,3,4].

Recently, the recycling of food waste has proven to be an attractive area of research for nutraceutical applications due to the high content of bioactive compounds contained in waste [5,6]. Several pharmaceutical companies are now thinking of agri-food by-products as alternative raw materials for the isolation of bioactive molecules to be included in nutraceuticals and food supplements [7]. Authorities, which are alarmed by air, soil, and water pollution generated by agricultural waste disposal, strongly encourage this recycling activity. Moreover, waste reuse presents several economic advantages: (i) it is cheap; (ii) abundant; (iii) enriched in bioactive molecules; as well as (iv) it receives the financial support of those governments promoting eco-compatible and pollution-reducing practices [8].

A significant portion of waste biomass is represented by agricultural by-products (e.g., leaves, bark, roots, seeds, and wood) [9], while another significant portion consists of second-best food products (fruits and vegetables) whose morphological and aesthetic characteristics do not meet the requirements of the modern world market [1,10].

Wine production, for example, one of the most important agro-industrial activities in the world, produces large amounts of biowaste. *Vitis* (*V.*) *vinifera* L., the most widely used species for wine production [11], produces tons of by-products including pomace, grape seeds, stalks, leaves, and shoots. This biowaste has already been largely used to obtain nutraceuticals and supplements enriched in bioactive compounds, especially polyphenols, molecules endowed with anti-inflammatory, antimicrobial, and antioxidant properties [7,12,13]. In order to produce new nutraceutical formulations, wine biowaste is also being chemically modified. Grape seeds, for example, rich in proanthocyanidins [6]—antioxidants endowed with great beneficial effect but of scarce bioavailability [7,8]—can be depolymerized under food-grade alkaline conditions to release more adsorbable flavan-3-ols. Indeed, in vitro data have shown for alkaline treated grape seed extracts, a better intestinal bioavailability and systemic absorption, with high antioxidant activity and beneficial healthy effects [9].

Biowaste matrices used for nutraceutical production might include also immature fruits derived from fruit thinning. This is a widespread agronomical procedure that consists of removing some of the small fruits produced by plants, allowing the remaining fruits to grow larger and reach the optimal standard-size for the market. Thinning may divest up to half the entire tree fruit load and results in massive agricultural waste production [14]. Thinned nectarines (*Prunus (P.) persica* L.) (TN) have, for example, aroused particular interest in the scientific literature as consequence of their high content of abscisic acid (ABA) [15], a plant phytohormone reaching its maximum concentration during the early stages of fruit development and involved in suppressing fruit ripening [16]. Consolidated evidence indicates ABA being a potent modulator of glucose homeostasis in humans [17]. Specifically, ABA has been shown to ameliorate glycaemic profile mainly through AMPK-mediated stimulation of peripheral glucose uptake [18]. This effect has been recently confirmed by in vivo studies showing that supplementation of nutraceuticals containing TN, significantly reduces glycaemic parameters in association with an insulin-sparing mechanism of action [19].

Although the Compendium of Botanicals mentions *P. persica* and *V. vinifera* as safe products to be included in nutraceuticals, functional foods, and food supplements, this list does not clearly refer to their waste by-products nor to the possibility of using chemically treated versions of them [20]. While initial controversies regarding a potential toxicity of wine biowaste have been solved, with *V. vinifera* L. grape pomace showing no toxicity [21,22], no toxicological data are available on alkaline treated biowaste extracts. Moreover, toxicological studies on formulations containing thinned fruit extracts, including thinned nectarines, are not available. The main safety concern in the reutilization of biowaste is that the unconventional parts of the plant used for the formulation could contain endogenous molecules that, alone or in synergism with others, could exert a toxic effect on the consumers [23]. A second concern about recycling is that biowaste, more than main fruits, could retain traces of pollutants used for cultivation (e.g., polycyclic aromatic hydrocarbons, aromatic amines, quinolines, pyridines, nitroquinolines and hydroquinone) [24].

Thus, while EFSA strongly encourages biowaste recycling, it invites producers to verify the biosafety of every substantially new product on the market. Among the toxicological tests, EFSA suggests using an in-vitro platform to confirm the non-genotoxicity of new products or formulations. Genotoxicity refers to the ability of a specific substance to modify the genome of the cells by causing DNA mutations or chromosomal recombination and rearrangements. Known genotoxic substances lead to various human diseases, promote cell transformation and cancer, amongst other effects [25]. DNA damage can indeed be considered a surrogate endpoint for carcinogenicity, since the latter occurs in mammals as a consequence of the accumulation of mutations [26].

Here, following EFSA advice, the Ames test (OECD 471) [27] and the micronucleus test (OECD 487) [28] were used to assess for the first time the mutagenicity of two nutraceuticals obtained from thinned nectarine (TN) (*P. persica* L., *var. nucipersica*) and alkaline treated grape seed extract (ATGSE) (*V. Vinifera* L.) waste biomasses.

## 2. Materials and Methods

### 2.1. Nutraceuticals

#### 2.1.1. Thinned Nectarines (TN)

TN of (*P. persica* (L.) Batsch var. *nucipersica*) were supplied by the orchards of the company “Giaccio Frutta” (Vitulazio, Caserta, Italy, 41°10′ N–14°13′ E), 20–25 days after full bloom, coinciding with the stage of fruit thinning. A total of 10 kg of whole fruits were frozen at −80 °C, freeze-dried, and ground to obtain 1 kg of a homogeneous powder. As already described by previous works [29], the quantitative polyphenolic composition of freeze-dried TN was represented by (µg/g ± standard deviation): caffeic acid 15.85 ± 0.06; catechin 128.32 ± 0.36; chlorogenic acid 1496.85 ± 0.22; epicatechin 34.63 ± 0.83; ferulic acid 10.59 ± 0.02; gallic acid 168.31 ± 1.51; kaempferol-3-***O***-glucoside 63.65 ± 3.01; naringenin 10.92 ± 0.42; neochlorogenic acid 1456.98 ± 1.19; *p*-coumaric acid 5.05 ± 0.33; procyanidin B1 + procyanidin B3 8.41 ± 0.02; procyanidin B2 6.55 ± 0.01; procyanidin C1 12.66 ± 0.01; quercetin 17.89 ± 0.41; quercetin-3-*O*-glucoside 166.01 ± 3.35; rutin 48.86 ± 0.67; syringic acid 115.16 ± 0.21; vanillic acid 19.28 ± 0.91.

#### 2.1.2. Grape Seed Extract (GSE) and Alkaline Treatment of GSE (ATGSE)

GSE from *V. vinifera* L. was a commercial extract titrated to 95% proanthocyanidins purchased from MB-Med S.r.l (Turin, Italy). The overall qualitative composition of the extract has already been described [30]. The percentage composition corresponds to: monomeric proanthocyanidins (49%), dimeric proanthocyanidins (24%), trimeric proanthocyanidins (9%), polymeric proanthocyanidins DP > 4 (4%), and galloylated proanthocyanidins (14%). In order to obtain a more bioavailable product, the GSE was subjected to a depolymerization method under alkaline conditions. To this end, 0.5 g of the sample was weighed into a centrifuge tube, to which 20 mL of distilled water and 600 µL of NaOH 1N were added. The tube was then placed in an orbital shaker (200 rpm) that stood in an incubator set at 45 °C for 4 h. The samples were then frozen at −80 °C and freeze-dried. The percentage composition of the sample obtained from the depolymerization process, named ATGSE, was as follows: monomeric proanthocyanidins (58%), dimeric proanthocyanidins (31%), trimeric proanthocyanidins (6%), polymeric proanthocyanidins DP > 4 (1%), galloylated proanthocyanidins (4%).

### 2.2. Chemicals and Reagents

Ampicillin (code 26-810), tetracycline (code 26-811), crystal violet (code 26-813), benzo(a)pyrene (BAP. CAS 50-32-8. code 60-114.6. LOT. NO 8197BP), sodium azide (NaN3, code 60-103.1), 2-aminoanthracene (2AA, code 60-107.21), 2-nitrofluorene (2-NF, code 60-111), 4-nitroquinoline-N-oxide (4-NQO, code 60-121.3), 9-aminoacridine (9AA, code 60-147.5), mitomycin C (Mit C. CAS Number 50-07-7. LOT.NO 0611718-2) were all purchased from Trinova Biochem GmbH (Geissen. Germany) as well as MutazymeTM, 10%, lyophilized rat liver S9 mix (20 mL/vial, code 11-402L). When indicated chemicals were dissolved in sterile dimethyl sulfoxide.

### 2.3. Ames Test

Genotoxic activity was evaluated by the bacterial reversion test (Ames test), using *Salmonella (S.) typhimurium* strains TA98, TA100, TA1535, TA1537 and *Escherichia (E.) Coli* strain WP2 trp UvrA, in the presence and absence of exogenous metabolization (S9). The method used followed OECD 471 guidelines [31]. Experiments were performed in triplicates and results are presented as mean mutagenic index ± SD.

#### 2.3.1. Bacteriological Media

Minimal glucose agar plates: Agar (15 g/L), Vogel–Bonner salts (MgSO_4_ × 7H_2_O (200 mg/L), citric acid × H_2_O 2 g/L, KH_2_PO_4_ 10 g/L, (NH_4_) NaHPO_4_ × 4H_2_O (3.5 g/L)), D-glucose (4.0 g/L), pH 7.0. Oxoid agar plates: Agar (15 g/L), Vogel–Bonner salts (MgSO_4_ × 7H_2_O (200 mg/L), citric acid × H_2_O 2 g/L, KH_2_PO_4_ 10 g/L. (NH_4_) NaHPO_4_ × 4H_2_O (3.5 g/L)), D-glucose (2.0 g/L), oxoid No.2 broth (25 g/L), pH 7.0. Top agar: Agar (7 g/L), NaCl 5 g/L, L-histidine HCl (10.4 mg/L), L-tryptophan HCl (10.1 mg/L), D-biotin (12.2 mg/L), pH 7.0. Media were steam sterilized at 15 lbs/sq for 120 min at 121 °C.

#### 2.3.2. Bacterial Growth and Storage

Bacterial cultures were grown at a temperature of 37 °C in 50 mL falcons with 9 volumes of air per volume of broth, as previously described [21]. Falcon tubes were placed in an incubator and shaken at 230 rpm. Cultures were grown overnight (approximately 109 cells per mL. Optical Density at λ = 600 nm of 1.0 ± 0.1). Bacterial strains were freshly defrosted each time.

#### 2.3.3. Bacterial Strains

The five bacterial strains *S. typhimurium* TA1535 (LOT. NO 5294D), *S. typhimurium* TA1537 (LOT NO. 5295D), *S. typhimurium* TA98 (LOT NO. 5293D), *S. typhimurium* TA100 (LOT NO. 5325D) and *E. coli* WP2 trp UvrA were obtained from Trinova Biochem (Giessen, Germany). For *S. typhimurium* TA98 strain, phenotype confirmation was performed as previously described [21], by growing an overnight culture in LB media to challenge 1–2 × 108 bacteria as follows: (a) L-His negative phenotype is confirmed by the absence of colonies on agar minimal plate without L-His; (b) the rfa phenotype is confirmed when the strain manifested zonal growth inhibition on LB agar plates in the presence of a 10 µg crystal violet disc (c) the presence of the R-factor plasmid is confirmed when the strain grew on LB agar plates containing 2 µg ampicillin disc (d) the absence of the pAQ1 plasmid is confirmed when the strain did not grow on agar minimal plates containing L-His, 2 µg ampicillin and 1 µg tetracycline disc.

For *S. typhimurium* TA100 strain, phenotype confirmation was performed by growing an overnight culture in LB media to then challenge 1–2 × 108 bacteria as follows: (a) L-His negative phenotype is confirmed by the absence of colonies on agar minimal plates without L-His; (b) the rfa phenotype is confirmed when the strain manifested zonal growth inhibition on LB agar plates in the presence of a 10 µg crystal violet disc (c) the presence of the R-factor plasmid is confirmed when the strain grew on LB agar plates containing a 2 µg ampicillin disc (d) the absence of the pAQ1 plasmid is confirmed when the strain did not grow on agar minimal plates containing L-His, 2 µg ampicillin and 1 µg tetracycline disc.

For *S. typhimurium* TA1535 strain, phenotype confirmation was performed by growing an overnight culture in LB media to then challenge 1–2 × 108 bacteria as follows: (a) L-His negative phenotype is confirmed by the absence of colonies on agar minimal plates without L-His; (b) the rfa phenotype is confirmed when the strain manifested zonal growth inhibition on LB agar plate in the presence of a 10 µg crystal violet disc (c) the absence of the R-factor plasmid is confirmed when the strain did not grow on LB agar plates containing a 2 µg ampicillin disc (d) the absence of the pAQ1 plasmid is confirmed when the strain did not grow on agar minimal plates containing L-His, 2 µg ampicillin and 1 µg tetracycline disc.

For *S. typhimurium* TA1537 strain, phenotype confirmation was performed by growing an overnight culture in LB media to then challenge 1–2 × 108 of bacteria as follows: (a) His negative phenotype is confirmed by the absence of colonies on agar minimal plates without L-His; (b) the rfa phenotype is confirmed when the strain manifested zonal growth inhibition on LB agar plates in the presence of a 10 µg crystal violet disc (c) the absence of the R-factor plasmid is confirmed when the strain did not grow on LB agar plates containing a 2 µg ampicillin disc (d) the absence of the pAQ1 plasmid is confirmed when the strain did not grow on agar minimal plates containing L-His, 2 µg ampicillin and 1 µg tetracycline disc.

For *E. coli* WP2 trp UvrA strain, phenotype confirmation was performed by growing an overnight culture in LB media to then challenge 1–2 × 108 of bacteria as follows: (a) trp- phenotype is confirmed when the strain did not grow on agar minimal plates in the absence of L-Trp; (b) the absence of the R-factor plasmid pKM101 is confirmed when the strain did not grow on LB agar plates containing 2 µg ampicillin.

#### 2.3.4. Metabolic Activation

Metabolic activation of nutraceuticals was performed by exogenous metabolization using S9 post-mitochondrial fraction as previously described [21]. S9 (code 11–402L. LOT NO. 4026) prepared from livers of Sprague Dawley male rats treated with Aroclor 1254 (500 mg/Kg i.p.). Lyophilized S9 was purchased from Trinova Biochem already supplemented with glucose-6-phosphatedehydrogenase (180 mg/mL), nicotinamide adenine dinucleotide phosphate (25 mg/mL), and potassium chloride (150 mM) mixed in the ratio 2:1:1:1. S9 was reconstituted in 20 mL of deionized water and stored at −80 °C. To prove S9 was able to activate pro-mutagens, we measured the number of revertant colonies of TA 100 and TA1535 strains growing in the presence of S9 and of BAP and 2AA. The TA 100 strain gave 386 colonies in the presence of BAP and TA1535 yielded 250 colonies in the presence of 2AA, respectively. The final concentration of S-9 fraction in the test system was 7% *v*/*v*.

#### 2.3.5. Nutraceutical Test Conditions

Since water is inert, it was used as a vehicle for all experiments. Stock solutions of the two nutraceuticals at the concentration of 1 mg/mL were freshly prepared in water as previously described [21]. The recommended maximum test concentration for soluble non-cytotoxic substances is 5 mg/plate. Test dilutions were obtained by diluting stock solutions in water. We tested eight dilutions for each nutraceutical (0.0016, 0.005, 0.016, 0.05, 0.16, 0.5, 1.6. and 5 mg/10 cm plate). At doses up to 5 mg/plate and on LB agar plates, none of the nutraceuticals caused growth inhibition of the tested bacterial strains, confirming that in the dilution range assayed here, all tested nutraceuticals were not cytotoxic to the bacteria strains (Table 1 and Table 2). Negative controls consisted of 100 µL water. Positive controls consisted: for *S. typhimurium* TA100, NaN3 1.25 µg/10 cm plate in the absence of S9 and BAP 3.0 µg/10 cm plate in the presence of S9; for *S. typhimurium* TA98, 2NF 1.0 µg/10 cm plate in the absence of S9 and BAP 3.0 µg/10 cm plate in the presence of S9; for *S. typhimurium* TA1535, NaN3 1.25 µg/10 cm plate in the absence of S9 and 2AA 1.0 µg/10 cm plate in the presence of S9; for *S. typhimurium* TA1537, 9AC 25.0 µg/10 cm plate in the absence of S9 and 2AA 1.0 µg/10 cm plate in the presence of S9; for *E. coli* WP2 trp UvrA, NQO 1.0 µg/10 cm plate in the absence of S9 and 2AA 10.0 µg/10 cm plate in the presence of S9.

#### 2.3.6. Experimental Procedure

The plate incorporation method was used as previously described [21]. Briefly, 5 mL of test solutions, 0.1 mL of fresh bacterial culture containing 108 viable cells and either 0.5 mL of water, or 0.5 mL of S9, were mixed with 2.0 mL of top agar. For the assay with metabolic activation, 0.5 mL of metabolic activation mixture containing 7% post-mitochondrial fraction. The contents of each tube were mixed and poured over the surface of a minimal agar plate. The overlay agar was allowed to solidify before incubation. Experiments were performed in triplicate for each condition. Plates were incubated at 37 °C for 72 h. After the incubation period, the number of revertant colonies per plate were counted.

### 2.4. Antimutagenicity Test

The experimental procedure for the antimutagenicity test followed a modified Ames assay. Briefly, to verify the ability of TN or ATGSE to counteract or reduce the genotoxicity of known mutagens, bacteria were cultured in the presence of known mutagens and of nutraceuticals (for *S. typhimurium* TA100 NaN3; for *S. typhimurium* TA98 2NF; for *S. typhimurium* TA1535 NaN3; for *S. typhimurium* TA1537 9AC; for *E. coli* WP2 trp UvrA NQO).

### 2.5. Micronucleus Test

The murine tumour cell line B16 and the human hepatoma cell line HuH-7 were grown in Dulbecco’s Modified Eagle’s Medium (DMEM) (supplemented with 10% FBS, streptomycin, penicillin and glutamine). B16 and HuH-7 were incubated at 37 °C in a 5% CO_2_ atmosphere with 100% humidity and sub-cultivated every two days to avoid over-confluency. For the micronucleus assay [28] cells were plated (2500 cells/well) in 96-well plates. At 24 h after plating, the culture medium was supplemented with three concentrations (100, 50, 25 µg/well) of nutraceuticals. At 6 h after treatment, nutraceuticals were removed, and media replaced. After 48 h from the wash out, cells were fixed in 3.7% formaldehyde for 30 min, permeabilized with 0.1% Triton-X100 and their nuclei stained with DAPI. Finally, the number of micronuclei were counted. Experiments were performed in triplicate and results are presented as mean percentage of cell presenting micronuclei ± SD.

### 2.6. Statistical Analysis

Statistical analyses were performed with GraphPad Prism 8.4.3 by using the *Student’s t-test*. *p*-values less than 0.05 were regarded as statistically significant.

## 3. Results

### 3.1. Ames Test

The Ames test [32] identifies genotoxic molecules by measuring the rate of mutations occurring in bacterial genomes upon exposure to a test chemical. The bacterial strains used in this assay (*S. typhimurium* and *E. coli*) present mutations in genes required for the synthesis of a specific amino acid (His in *Salmonella typhimurium* and Trp in *Escherichia coli*). As a consequence of these mutations, these bacteria have lost autotrophy for these amino acids and stop growing in their absence [33]. Genotoxic chemicals revert-back the mutated genes of the bacterial strains to the wt sequences, allowing the growth of revertant colonies even in the absence of amino acids. To identify chemicals acquiring genotoxicity upon in vivo metabolization, substances are tested in the presence of a metabolic activation system derived from rodent liver microsomes and referred to as S9 [34]. A mutagenic index (the ratio between the number of revertants per plate after treatment with the nutraceuticals and the number of revertants per plate obtained after treatment with the negative control (vehicle)) is then calculated. Known mutagens (BAP, NaN3, 2AA, 2-NF, 4-NQO, 9AA), are included in the assay as positive controls. A compound is defined mutagenic if it causes a two-fold increase in the mutagenic index.

The experimental platform started with a preliminary experiment. The AMES test is incompatible with the testing of antibiotic molecules. Genotoxic nutraceuticals endowed with antibiotic or cytotoxic activity would indeed reduce the number of revertant colonies giving erroneously safe results according to the Ames test. In order to test if, in the range of concentrations assayed, TN and ATGSE would have displayed antibiotic effects, four *S. Tiphymurium* strains TA98, TA100, TA1535, TA1537 and the *E.Coli* WP2 trp UvrA strain were plated on Luria Broth (LB) agar plates (a rich growth-medium containing sources of His and Trp) and supplemented with increasing doses of the nutraceuticals. As shown in Table 1 and Table 2, in the range of concentrations tested, TN or ATGSE did not reduce or inhibit the growth of the five bacterial strains tested.

Table 3, Table 4, Table 5 and Table 6 show the results of the Ames test for TN and ATGSE. The tables indicate the number of revertant colonies of each bacterial strain (*E.Coli* WP2 trp UvrA, *S. Tiphymurium* TA98, TA100, TA1535, TA1537) grown on minimal agar plates (not containing His or Trp) after incubation with one of the eight different concentrations of TN (Table 3) and ATGSE (Table 4). Treatments were performed both in the presence (+S9) and in the absence (−S9) of metabolic activation to mimic hepatic metabolization, as for EFSA indications. In Table 5 and Table 6, the mutagenic index of TN (Table 5) and ATGSE (Table 6) were calculated and compared with the mutagenic index of known genotoxic and carcinogenic molecules.

As shown in Table 3, Table 4, Table 5 and Table 6, in the presence or in the absence of metabolic activation (S9), both nutraceuticals are non-genotoxic for any of the bacterial strains at any of the concentrations tested.

### 3.2. Antimutagenicity Test

Interestingly, the number of revertant colonies grown in the presence of nutraceuticals were less than those grown in the presence of the vehicle. The reduction in growth followed a dose–response curve, with bacterial growth reducing at higher doses of nutraceuticals. However, as shown in Table 1 and Table 2, when LB was used as growth medium TN and ATGSE did not exert an antibiotic effect at the concentrations tested. A likely explanation for this growth inhibition on minimal agar plates would be an anti-mutagenic potential of TN and ATGSE. Polyphenols contained in plant extracts have been already shown to protect cells from mutations, either by chemically shielding them from mutagens or by improving the cellular DNA-damage response or proof-reading capacity of DNA polymerases [35]. To test this hypothesis, *S. tiphymurium* and *E.Coli* strains were treated with known mutagens in the presence of either TN or ATGSE. As shown in Table 7, a reduction in the mutagenic index of these mutagens was measured in the presence of the two nutraceutical products, confirming the antimutagenic potential of TN and ATGSE.

### 3.3. Micronucleus Test

Micronuclei are small round-shaped chromosomic fragments (bodies), usually present next to the main nucleus in the cytoplasm of cells. Micronuclei can physiologically occur in cells, but their number increases drastically when cells are exposed to substances that cause structural and numerical chromosomal alterations [36]. The micronucleus test assay allows the detection of micronuclei in different types of cells, such as human and rodent cells. B16 murine melanoma cells and HuH7 human hepatoma cells were used for the assay by virtue of their tendency to generate high numbers of micronuclei. As a positive control the alkylating agent cisplatin was used. As shown in Table 8, and compared to vehicle, none of the compounds increased the number of micronuclei in B16 or in HuH7 cells.

## 4. Discussion

Nutraceuticals produced from recycled biowaste are becoming popular over the counter products. However, those that have been risk-assessed in terms of safety are rare. Thus, waste biomasses by virtue of their chemical complexity, could in many cases, undermine the overall safety of the final nutraceutical product. This especially applies to chemically modified food by-products, that as consequence of the reactions, could have generated harmful molecular species. Developers and producers of nutraceuticals are thus advised to assess the safety of their final nutraceutical products, in compliance with EFSA regulations. In most cases, the genotoxic potential of a nutraceutical is considered as the resulting sum of the genotoxicity of its components. Each component is thus evaluated independently from the others and is only considered unsafe if its amount is higher than dosages reported as toxic, lethal or causing side effects. However, this approach does not take into account that in the final products, each component could synergistically or antagonistically influence the others, and alter the overall toxicity of the nutraceutical as well as its pharmacokinetic parameters (bioavailability, bioaccessibility, bioactivity). Authorities are thus advising testing the safety of new nutraceutical formulations, especially those obtained from new or alternative matrices.

To the best of our knowledge, the mutagenicity of TN and ATGSE has never been assessed. Using the Ames test, Yamomoto et al. have proved the antimutagenic effect of a hexane extract of Persicae peach semen (*P. persica Bat*), and shown this extract was able to inhibit the mutagenicity induced by the genotoxic molecule BAP [37]. Unprocessed wine and grape pomace extracts were shown to be non-genotoxic in the Ames test up to 5 mg/plate [21]. Grape seeds were shown to be genotoxically safe up to 200 μg/mL using the micronucleus test [22].

The remaining information on the biosafety of TN and ATGSE relates to their pure components. Pure ABA has been tested by Ames Test on six *S. typhimurium* strains (TA98, TA100, TA 1535, TA 1537) and on the *E. coli* strain WP2uvrA [38] and proven to be non-genotoxic up to a concentration of 5 mg/plate. However, the assay was performed without metabolic activation. The genotoxic safety of pure monomeric, dimeric, trimeric and polymeric procyanidins have been confirmed using the micronucleus test in murine bone marrow up to the concentration of 300 mg/mL [39]. The information available for quercetin is discordant. The polyphenol has shown to be genotoxic in the Ames test [40,41], the chromosome aberration test [42], and the micronucleus test [43]. Quercetin and rutin, however, were proven to be genotoxically safe up to the concentration of 300 mg/mL [39]. More recently, quercetin has been shown to be non-genotoxic in the Ames test up to a concentration of 5 mg/plate [44] and according to the micronucleus test in mice and Wister rats, up to the concentration of 2 g/kg [45]. Divergent results have also been presented for caffeic acid: (i) on cultured lymphocytic HL-60 and Jurkat cells, the molecule shows no genotoxicity up to 100 µM [46], however, (ii) it has genotoxic effects on rat hepatoma HTC cells at concentrations of 500 and 1500 μM [47]. Vanillic acid has shown genotoxicity in lymphocytes collected from healthy donors at the concentration of 2 µg/mL [48]. Kaempferol was shown to have mutagenic activity both in Ames and chromosome aberration tests [41]. Gallic acid has shown no toxicity in mice up to the concentration of 400 mg/kg of body weight [49].

Here, following EFSA advice to confirm the non-genotoxicity of final nutraceutical products, the Ames test (OECD 471) and the micronucleus test (OECD 487) were used to confirm the genotoxic safety of two nutraceuticals obtained from TN (*P. persica* L.) and ATGSE (*V. Vinifera* L.) waste biomasses. The results presented here show that these nutraceuticals are genotoxically safe. The two assays we used both present limitations. The Ames test uses bacterial DNA as a mimic of the human genome, without taking into account that the latter contains introns and is compacted by histones, and can thus be differently affected by mutagens. Moreover, silent mutations (i.e., not altering the primary sequence of proteins) or mutations occurring at promoter regions could still result in the activation of oncogenes and in the repression of oncosuppressors and be similarly dangerous for human cells rather than classic point mutations detected by the Ames test. On the other hand, the micronucleus test identifies only massive alterations in chromosomes while missing minimal, but still potentially dangerous, chromosomal rearrangements. Despite these limitations the two tests remain the gold standards for mutagenicity risk assessment and are suggested by EFSA and other international agencies.

Interestingly, both TN (*P. persica* L.) and ATGSE (*V. Vinifera* L.) act as anti-mutagens, reproducing the effects of their respective main fruits [50]. This is likely the result of their bioactive fractions being enriched in bioactive compounds, especially polyphenols, present in higher quantities compared to full-harvest fruits [51]. In ATGSE, the food-grade alkali treatment promotes a 10% increase in monomeric flavan 3-ol and dimeric proanthocyanidins [30]. TN at the early stage of fruit development have indeed notably higher polyphenol content than ripe fruits, they are a source of ABA and present higher antioxidant potential [51]. In particular, a recent study highlighted the rich qualitative and quantitative composition of TN in terms of hydroxycinnamic acids, flavonols, flavanols, and procyanidins [51].

ATGSE, as a source of proanthocyanidins and TN, as sources of ABA and polyphenols, can be considered green, sustainable and valuable nutraceutical products. The results presented here on their safety, adds to the already available literature on these products and confirms their interest as new nutraceuticals.

## Figures and Tables

**Table 1:** Number of colonies of the indicated bacterial strains grown on LB plates in the presence of different concentrations of TN.

mg/Plate	TA98	TA100	TA1535	TA1537	WP2
5	441 ± 73 ^#^	409 ± 59 ^#^	497 ± 34 ^#^	1040 ± 39 ^#^	1054 ± 126 ^#^
1.6	590 ± 280 ^#^	430 ± 34 ^#^	467 ± 42 ^#^	1016 ± 84 ^#^	1043 ± 35 ^#^
0.5	452 ± 18 ^#^	414 ± 27 ^#^	506 ± 67 ^#^	974 ± 209 ^#^	990 ± 16 ^#^
0.16	393 ± 128 ^#^	407 ± 42 ^#^	450 ± 19 ^#^	979 ± 129 ^#^	1055 ± 196 ^#^
0.05	383 ± 154 ^#^	417 ± 54 ^#^	429 ± 37 ^#^	1078 ± 13 ^#^	1038 ± 139 ^#^
0.016	449 ± 32 ^#^	415 ± 119 ^#^	455 ± 31 ^#^	1040 ± 28 ^#^	975 ± 23 ^#^
0.005	486 ± 25 ^#^	442 ± 81 ^#^	441 ± 36 ^#^	988 ± 94 ^#^	924 ± 56 ^#^
0.0016	504 ± 83 ^#^	376 ± 52 ^#^	453 ± 40 ^#^	1089 ± 27 ^#^	979 ± 25 ^#^
Antibiotic	5 ± 9 *	0 ± 0 *	0 ± 0 *	0 ± 0 *	0 ± 0 *
Negative Control	426 ± 80	389 ± 13	463 ± 7	1048 ± 27	987 ± 18

Negative controls consisted of 100 µL water. Positive controls consisted of 2 µg of ampicillin and 1 µg of tetracycline per plate. The data represent mean of three replicates. Data were analysed with the *Student’s t-test*; * *p* ≤ 0.05 indicates values significantly different from negative control; ^#^ indicates value not significantly different from negative control.

**Table 2:** Number of colonies of the indicated bacterial strains grown on LB plates in the presence of different concentrations of ATGSE.

mg/Plate	TA98	TA100	TA1535	TA1537	WP2
5	464 ± 36 ^#^	322 ± 106 ^#^	400 ± 77 ^#^	1136 ± 107 ^#^	1028 ± 150 ^#^
1.6	397 ± 67 ^#^	466 ± 116 ^#^	447 ± 23 ^#^	1062 ± 41 ^#^	1002 ± 35 ^#^
0.5	496 ± 35 ^#^	421 ± 15 ^#^	473 ± 8 ^#^	958± 135 ^#^	995 ± 9 ^#^
0.16	526 ± 99 ^#^	430 ± 83 ^#^	449 ± 14 ^#^	990 ± 122 ^#^	1082 ± 184 ^#^
0.05	427 ± 80 ^#^	379 ± 72 ^#^	433 ± 61 ^#^	1127 ± 30 ^#^	1037 ± 140 ^#^
0.016	487 ± 43 ^#^	404 ± 13 ^#^	443 ± 34 ^#^	1025 ± 97 ^#^	984 ± 22 ^#^
0.005	569 ± 81 ^#^	399 ± 36 ^#^	438 ± 22 ^#^	1219 ± 263 ^#^	917 ± 47 ^#^
0.0016	549 ± 151 ^#^	398 ± 9 ^#^	456 ± 41 ^#^	1049 ± 76 ^#^	987 ± 19 ^#^
Antibiotic	5 ± 9 *	0 ± 0 *	0 ± 0 *	0 ± 0 *	0 ± 0 *
Negative Control	426 ± 80	389 ± 13	463 ± 7	1048 ± 27	987 ± 18

Negative controls consisted of 100 µL water. Positive controls consisted of 2 µg of ampicillin and 1 µg of tetracycline per plate. The data represent mean of three replicates. Data were analysed with the *Student’s t-test*; * *p* ≤ 0.05 indicates value significantly different from negative control; ^#^ indicates value not significantly different from negative control.

**Table 3:** Number of revertant colonies/plate grown upon treatment of the indicated bacteria with TN.

			**−S9**		
**mg/Plate**	**TA98**	**TA100**	**TA1535**	**TA1537**	**WP2**
5	51 ± 9 ^#^	37 ± 4 ^#^	34 ± 3 ^#^	111 ± 17 ^#^	33 ± 1 ^#^
1.6	59 ± 16 ^#^	47 ± 8 ^#^	38 ± 21 ^#^	82 ± 13 ^#^	37 ± 5 ^#^
0.5	51 ± 3 ^#^	29 ± 17 ^#^	15 ± 9 ^#^	36 ± 14 ^#^	20 ± 5 ^#^
0.16	32 ± 4 ^#^	21 ± 15 ^#^	23 ± 4 ^#^	23 ± 1 ^#^	24 ± 3 ^#^
0.05	56 ± 4 ^#^	31 ± 24 ^#^	33 ± 2 ^#^	36 ± 16 ^#^	38 ± 2 ^#^
0.016	66 ± 14 ^#^	52 ± 23 ^#^	48 ± 10 ^#^	42 ± 9 ^#^	31 ± 4 ^#^
0.005	75 ± 23 ^#^	59 ± 3 ^#^	62 ± 6 ^#^	55 ± 20 ^#^	50 ± 9 ^#^
0.0016	99 ± 1 ^#^	81 ± 4 ^#^	85 ± 20 ^#^	59 ± 7 ^#^	100 ± 6 ^#^
Positive Control	396 ± 12 *	1043 ± 7 *	764 ± 11 *	892 ± 14 *	468 ± 13 *
Negative Control	104 ± 15	80 ± 12	89 ± 15	160 ± 14	205 ± 18
			**+S9**		
**mg/plate**	**TA98**	**TA100**	**TA1535**	**TA1537**	**WP2**
5	64 ± 5 ^#^	58 ± 8 ^#^	49 ± 16 ^#^	190 ± 8 ^#^	46 ± 4 ^#^
1.6	76 ± 8 ^#^	97 ± 8 ^#^	51 ± 2 ^#^	89 ± 14 ^#^	62 ± 7 ^#^
Positive Control	n.t.	384 ± 8 *	248 ± 8 *	n.t.	n.t.
Negative Control	114 ± 9	78 ± 4	129 ± 4	212 ± 12	258 ± 6

Negative controls consisted of 100 µL water. Positive controls consisted of: NaN3 (−S9) and BAP (+S9 for *S. typhimurium* TA100); 2NF (−S9) for *S. typhimurium* TA98; NaN3 (−S9) and 2AA (+S9) for *S. typhimurium* TA1535; 9AC (−S9) for *S. typhimurium* TA1537; NQO (−S9) for *E. coli* WP2 trp UvrA. (n.t.= not tested). The data represent the mean of three replicates. Data were analysed with the *Student’s t-test*; * *p* ≤ 0.05 indicates value significantly different from negative control; ^#^ indicates value not significantly higher than negative control.

**Table 4:** Number of revertant colonies/plate grown upon treatment of the indicated bacteria with ATGSE.

			**−S9**		
**mg/Plate**	**TA98**	**TA100**	**TA1535**	**TA1537**	**WP2**
5	44 ± 6 ^#^	59 ± 6 ^#^	59 ± 28 ^#^	62 ± 4 ^#^	34 ± 5 ^#^
1.6	22 ± 2 ^#^	37 ± 6 ^#^	22 ± 2 ^#^	43 ± 16 ^#^	42 ± 23 ^#^
0.5	23 ± 8 ^#^	15 ± 13 ^#^	11 ± 7 ^#^	40 ± 12 ^#^	24 ± 27 ^#^
0.16	23 ± 3 ^#^	4 ± 16 ^#^	4 ± 1 ^#^	17 ± 16 ^#^	16 ± 4 ^#^
0.05	20 ± 15 ^#^	33 ± 16 ^#^	15 ± 16 ^#^	30 ± 5 ^#^	19 ± 22 ^#^
0.016	33 ± 6 ^#^	44 ± 3 ^#^	37 ± 8 ^#^	37 ± 10 ^#^	39 ± 6 ^#^
0.005	69 ± 5 ^#^	76 ± 7 ^#^	81 ± 11 ^#^	64 ± 17 ^#^	69 ± 17 ^#^
0.0016	109 ± 6 ^#^	79 ± 10 ^#^	92 ± 15 ^#^	47 ± 9 ^#^	113 ± 7 ^#^
Positive Control	382 ± 6 *	1049 ± 6 *	782 ± 13 *	896 ± 5 *	461 ± 7 *
Negative Control	90 ± 15	88 ± 8	88 ± 13	171 ± 1	204 ± 10
			**+S9**		
**mg/plate**	**TA98**	**TA100**	**TA1535**	**TA1537**	**WP2**
5	64 ± 5 ^#^	58 ± 8 ^#^	49 ± 16 ^#^	190 ± 7 ^#^	47 ± 3 ^#^
1.6	76 ± 8 ^#^	97 ± 7 ^#^	51 ± 2 ^#^	89 ± 14 ^#^	62 ± 8 ^#^
Positive Control	n.t.	384 ± 8 *	248 ± 8 *	n.t.	n.t.
Negative Control	114 ± 9	78 ± 4	129 ± 4	212 ± 12	258 ± 6

Negative controls consisted of 100 µL water. Positive controls consisted of: NaN3 (−S9) and BAP (+S9) for *S. typhimurium* TA100; 2NF (−S9) for *S. typhimurium* TA98; NaN3 (−S9) and 2AA (+S9) for *S. typhimurium* TA1535; 9AC (−S9) for *S. typhimurium* TA1537; NQO (−S9) for *E. coli* WP2 trp UvrA. (n.t.= not tested). The data represent the mean of three replicates. Data were analysed with the *Student’s t-test*; * *p* ≤ 0.05 indicates value significantly higher than negative control; ^#^ indicates value not significantly higher than negative control.

**Table 5:** Mutagenic index for the indicated concentrations of TN.

			**−S9**		
**mg/Plate**	**TA98**	**TA100**	**TA1535**	**TA1537**	**WP2**
5	0.49 ± 0.07 *^,°^	0.47 ± 0.05 *^,°^	0.38 ± 0.04 *^,°^	0.70 ± 0.07 *^,°^	0.16 ± 0.01 *^,°^
Positive Control	4.21 ± 0.95 *	11.81 ± 0.6 *	8.7 ± 0.75 *	5.24 ± 0.03 *	2.27 ± 0.08 *
Negative Control	1.00± 0.14 ^°^	1.00 ± 0.07 ^°^	1.00 ± 0.12 ^°^	1.00 ± 0.06 ^°^	1.00 ± 0.05 ^°^
			**+S9**		
**mg/plate**	**TA98**	**TA100**	**TA1535**	**TA1537**	**WP2**
5	0.57 ± 0.04 *^°^	0.75 ± 0.07 *^°^	0.38 ± 0.07 *^,°^	0.90 ± 0.02 *^,°^	0.18 ± 0.09 *^,°^
Positive Control	1.30 ± 0.1 * [21]	4.20 ± 0.46 *	2.10 ± 0.13 *	1.30 ± 0.1 * [21]	1.30 ± 0.1 * [21]
Negative Control	1.00 ± 0.07 ^°^	1.00 ± 0.04^°^	1.00 ± 0.03 ^°^	1.00 ± 0.08 ^°^	1.00 ± 0.02 ^°^

Negative controls consisted of 100 µL water. Positive controls consisted of NaN3 (−S9) and BAP (+S9) for *S. typhimurium* TA100; 2NF (−S9) and BAP (+S9) [21] for *S. typhimurium* TA98; NaN3 (−S9) and 2AA (+S9) for *S. typhimurium* TA1535; 9AC (−S9) and 2AA (+S9) [21] for *S. typhimurium* TA1537; NQO (−S9) and 2AA (+S9) [21] for *E. coli* WP2 trp UvrA. The data represent the mean of three replicates. Data were analysed with the *Student’s t-test*; * *p* ≤ 0.05 indicates value significantly different from negative control. ^°^ *p* ≤ 0.05 indicates value significantly different from positive control.

**Table 6:** Mutagenic index for the indicated concentrations of ATGSE.

			**−S9**		
	**TA98**	**TA100**	**TA1535**	**TA1537**	**WP2**
ATGSE 5 mg	0.49 ± 0.08 *^,°^	0.67 ± 0.05 *^,°^	0.66 ± 0.20 *^,°^	0.36 ± 0.02 *^,°^	0.17 ± 0.02 *^,°^
Positive Control	4.21 ± 0.95 *	11.81 ± 0.6 *	8.7 ± 0.75 *	5.24 ± 0.03 *	2.27 ± 0.08 *
Negative Control	1.00± 0.14 ^°^	1.00 ± 0.07 ^°^	1.00 ± 0.12 ^°^	1.00 ± 0.06 ^°^	1.00 ± 0.05 ^°^
			**+S9**		
	**TA98**	**TA100**	**TA1535**	**TA1537**	**WP2**
ATGSE 5 mg	0.39 ± 0.04 *^,°^	0.56 ± 0.05 *^,°^	0.56 ± 0.02 *^,°^	0.41 ± 0.03 *^,°^	0.18 ± 0.02 *^,°^
Positive Control	1.30 ± 0.1 * [21]	4.20 ± 0.46 *	2.10 ± 0.13 *	1.30 ± 0.1 * [21]	1.30 ± 0.1 * [21]
Negative Control	1.00 ± 0.07 ^°^	1.00 ± 0.04 ^°^	1.00 ± 0.03 ^°^	1.00 ± 0.08 ^°^	1.00 ± 0.02 ^°^

Negative controls consisted of 100 µL water. Positive controls consist of NaN3 (−S9) and BAP (+S9) for *S. typhimurium* TA100; 2NF (−S9) and BAP (+S9) [21] for *S. typhimurium* TA98; NaN3 (−S9) and 2AA (+S9) for *S. typhimurium* TA1535; 9AC (−S9) and 2AA (+S9) [21] for *S. typhimurium* TA1537; NQO (−S9) and 2AA (+S9) [21] for *E. coli* WP2 trp UvrA. The data represent the mean of three replicates. Data were analysed with the *Student’s t-test*; * *p* ≤ 0.05 indicates value significantly different from negative control. ^°^ *p* ≤ 0.05 indicates value significantly different from positive control.

**Table 7:** Mutagenic index of genotoxic compounds in the presence or in the absence of 5 mg/plate of TN or ATGSE.

	TA98	TA100	TA1535	TA1537	WP2
Known Mutagen	3.83 ± 0.31 *^,°^	2.07 ± 0.12 *^,°^	2.30 ± 0.30 *^,°^	9.25 ± 0.23 *^,°^	5.01 ± 0.13 *^,°^
Known Mutagen + TN	1.54 ± 0.15 *^°^	0.85 ± 0.05 ^°^	1.02 ± 0.30 ^°^	1.20 ± 0.05 *^,°^	0.78 ± 0.10 *^,°^
Known Mutagen +ATGSE	0.80 ± 0.30 ^°^	0.85 ± 0.28 ^°^	1.33 ± 0.10 ^°^	0.57 ± 0.05 *^,°^	0.50 ± 0.4 *^,°^
Negative control	1.00 ± 0.05 ^°^	1.00 ± 0.03 ^°^	1.00 ± 0.06 ^°^	1.00 ± 0.03 ^°^	1.00 ± 0.05 ^°^

Negative controls consisted of 100 µL water. Positive controls consisted of NaN3 for *S. typhimurium* TA100; 2NF for *S. typhimurium* TA98; NaN3 for *S. typhimurium* TA1535; 9AC for *S. typhimurium* TA1537; NQO for *E. coli* WP2 trp UvrA. The data represent the mean of three replicates. Data were analysed with the *Student’s t-test*; * *p* ≤ 0.05 indicates value significantly different from negative control. ^°^ *p* ≤ 0.05 indicates value significantly different from known mutagen.

**Table 8:** Percentage of murine B16 melanoma and human HuH-7 hepatoma cells presenting micronuclei upon treatment with the indicated concentrations of TN and ATGSE.

		B16		HuH7
µg/Well	TN	ATGSE	TN	ATGSE
100	7.6 ± 1.0 #	6.9 ± 3.1 #	11.1 ± 0.5 #	2.3 ± 1.9 #
50	4.0 ± 5.1 #	5.1 ± 1.1 #	9.7 ± 3.1 #	4.9 ± 0.3 #
25	2.5 ± 1.1 #	9.5 ± 1.1 #	5.7 ± 0.7 #	1.4 ± 0.7 #
Negative control	9.0 ± 1.3 #	8.7 ± 1.2 #	10.9 ±1.0 #	11.0 ± 3.2 #
Positive control	31.4 ± 2.1 *	35.0 ± 1.9 *	33.5 ± 3.4 *	37.1 ± 5.0 *

Negative control consisted of unsupplemented DMEM, positive control consisted of cisplatin 30 µM. The data represent the mean of three replicates and correspond to the percentage of cells presenting micronuclei (*n* = 200 from ten randomly chosen image fields). Data were analysed with the *Student’s t-test*; * *p* ≤ 0.05 indicates value significantly higher than negative control; ^#^ indicates value not significantly higher than negative control.

## Data Availability

Data is contained within the article.
